# Genome-Wide Association Mapping of Grain Micronutrients Concentration in *Aegilops tauschii*

**DOI:** 10.3389/fpls.2019.00054

**Published:** 2019-02-07

**Authors:** Sanu Arora, Jitender Cheema, Jesse Poland, Cristobal Uauy, Parveen Chhuneja

**Affiliations:** ^1^School of Agricultural Biotechnology, Punjab Agricultural University, Ludhiana, India; ^2^John Innes Centre, Norwich Research Park, Norwich, United Kingdom; ^3^Department of Plant Pathology and Agronomy, Wheat Genetics Resource Centre, Kansas State University, Manhattan, KS, United States

**Keywords:** wild progenitors, *Aegilops tauschii*, micronutrients, GWAS, biofortification

## Abstract

Bread wheat is an important and the most consumed cereal worldwide. However, people with predominantly cereal-based diets are increasingly affected by micronutrient deficiencies, suggesting the need for biofortified wheat varieties. The limited genetic diversity in hexaploid wheat warrants exploring the wider variation present in wheat wild relatives, among these *Aegilops tauschii*, the wild progenitor of the bread wheat D genome. In this study, a panel of 167 *Ae. tauschii* accessions was phenotyped for grain Fe, Zn, Cu, and Mn concentrations for 3 years and was found to have wide variation for these micronutrients. Comparisons between the two genetic subpopulations of *Ae. tauschii* revealed that lineage 2 had higher mean values for Fe and Cu concentration than lineage 1. To identify potentially new genetic sources for improving grain micronutrient concentration, we performed a genome-wide association study (GWAS) on 114 non-redundant *Ae. tauschii* accessions using 5,249 genotyping-by-sequencing (GBS) markers. Best linear unbiased predictor (BLUP) values were calculated for all traits across the three growing seasons. A total of 19 SNP marker trait associations (MTAs) were detected for all traits after applying Bonferroni corrected threshold of -log_10_(*P*-value) ≥ 4.68. These MTAs were found on all seven chromosomes. For grain Fe, Zn, Cu, and Mn concentrations, five, four, three, and seven significant associations were detected, respectively. The associations were linked to the genes encoding transcription factor regulators, transporters, and phytosiderophore synthesis. The results demonstrate the utility of GWAS for understanding the genetic architecture of micronutrient accumulation in *Ae. tauschii*, and further efforts to validate these loci will aid in using them to diversify the D-genome of hexaploid wheat.

## Introduction

The global population is anticipated to cross the mark of 9.7 billion by 2050. Ensuring food and nutritional security to this population poses a huge challenge especially under impending climatic variability and resource scarcity. Adequate intake of nutritious food enriched with essential micronutrients is a prerequisite for humans to meet their metabolic needs and maintain good health. The term “micronutrients” refers to a broad list of minerals and vitamins that the body needs in adequate proportions to function properly. They play an important role in cell physiology as cofactors for proteins that carry out the fundamental biological functions (Tapiero et al., 2003). Some of the micronutrients are relatively scarce in common food sources, which can lead to their deficiencies in humans. People living in developing countries and who tend to rely heavily on cereal-based diets are particularly prone to suffer from micronutrient deficiencies, a phenomenon often termed as “hidden hunger” (Khush et al., 2012). Iron (Fe) deficiency is the most prevalent nutritional disorder in the world affecting 2 billion people worldwide and suboptimal zinc (Zn) nutrition is more common than previously believed (Stoltzfus and Dreyfuss, 1998; World Health Organiztion, 2006). These deficiencies may cause several physiological disorders, including impaired mental and physical development, anemia, tissue hypoxia, stunting, and blindness (Stevens et al., 2013).

Several strategies including food fortification, supplementation, and dietary diversification have been implemented to fight these deficiencies. However, the need to have a more sustainable and cost-effective solution continues to be pursued globally. Biofortification of existing crops, that is, the development of nutritionally enriched crop varieties, is one of the most powerful tools to address micronutrient malnutrition. It uses conventional breeding and/or biotechnology approaches to increase the micronutrient content in the edible part of staple crops. Wheat is one of the most important cereal crops serving as a staple food source for 30% of the human population. It provides up to 60% of the daily calories intake especially for people living in developing countries. Therefore, the nutritional quality of wheat has a significant impact on overall human health worldwide. Cultivated wheat, however, contains sub-optimal quantities of micronutrients with the majority of Fe and Zn localized to the seed aleurone and embryo, which are removed during milling. In different studies, the range of these micronutrients in wheat was reported between 28.8–50.8 mg/kg for Fe, 13.5–34.5 mg/kg for Zn (Zhao et al., 2009), 24–28 mg/kg for Mn, and 3.5–4.4 mg/kg for Cu (Suchowilska et al., 2012), while the HarvestPlus has established target levels of 52 and 33 mg/kg for Fe and Zn (Bouis and Welch, 2010), which is higher than or closer to the upper range of the aforementioned values.

Genetic biofortification of wheat varieties using both classical breeding approaches to characterize germplasm for mineral variability and marker-assisted selection (MAS) using gene-based markers can enhance the micronutrient content of the edible part as well as their bioavailability (Khush et al., 2012). However, a major bottleneck for wheat biofortification is the genetic erosion during domestication which limited the genetic variability for Fe and Zn in the cultivated wheat gene pool. The genetic variation of these micronutrients in wild wheat progenitors offers a potentially rich resource for the future genetic improvement of wheat nutritional value. The wild relatives of hexaploid wheat include *Aegilops tauschii*, *Triticum boeoticum*, *Triticum monococcum*, *Triticum dicoccoides*, *Aegilops kotschyi*, *Aegilops longissima*, and *Aegilops speltoides*, and have been reported among the most promising sources of high Fe and Zn grain concentration (Cakmak et al., 2000; Chhuneja et al., 2006; Rawat et al., 2009).

*Ae. tauschii* is an attractive resource for improving the genetic variability of micronutrients in cultivated wheat as it can recombine with the D-genome of hexaploid wheat. *Ae. tauschii* is a diploid (2*n* = 14, DD), self-pollinating (cleistogamic) goatgrass species in the Triticeae tribe of the grass family. It consists of two phylogenetic lineages, designated as L1 and L2, broadly associated with ssp. *tauschii* and ssp. *strangulata*, respectively (Wang et al., 2013). Using *Ae. tauschii* for biofortification requires an understanding of the genetic architecture of mineral nutrient accumulation in the grains. Mineral accumulation is a complex quantitative trait controlled by multiple genes and greatly affected by genetic × environment interactions. Therefore, it is important to dissect the genetic basis of variability governing Fe and Zn concentrations in the grains in order to exploit this variability in the development of micronutrient enriched cultivars.

Most genetics studies undertaken in wheat have used linkage mapping to study the genetic basis of micronutrient accumulation. This involves establishing linkage disequilibrium (LD) in populations derived from bi-parental crosses to identify genes/QTLs associated with the trait of interest. However, due to restricted number and position of meiotic events, the resolution of QTL mapping is often confined to 10–30 cM and it can analyze only a small fraction of total possible alleles that exist in the population from which the parents originated (Zhu et al., 2008). In contrast, association mapping (AM) offers an alternative to linkage mapping and can help identify alleles represented in a broader set of germplasm (Yu and Buckler, 2006). In this study, we report the investigation of the loci controlling accumulation of four micronutrients (Fe, Zn, Cu, and Mn) in *Ae. tauschii* germplasm through genome wide association studies (GWASs).

## Materials and Methods

### Plant Material

A set of 167 *Ae. tauschii* accessions maintained at the Wheat Germplasm Collection, Punjab Agricultural University (PAU), Ludhiana (30° 52′N, 75° 56′E), were used in this study and the detailed information of these accessions was provided in Arora et al. (2017). Two bread wheat cultivars, PBW343 and WL711, were included in the study as reference checks for phenotypic variation observed in the *Ae. tauschii*.

### Grain Digestion and Micronutrient Evaluation

During the normal cropping season, *Ae. tauschii* accessions were grown at PAU, Ludhiana, for three consecutive seasons with recommended agronomic practices. Each accession was planted in a single row of 2 m length with 0.7 m spacing between the rows. The spikes were harvested at maturity and stored in glassine bags. Precautions were taken to avoid any metallic or dust contamination of grains while harvesting and analyzing. For each accession the grains were divided into three parts and analyzed as three replicates for Fe, Zn, Cu, and Mn concentrations using simultaneous multi-element inductively coupled plasma–optical emission spectrometer (ICP-OES, Perkin Elmer). Briefly, the whole grain samples were quickly washed with distilled water to remove any surface contamination and dried in hot air oven at 50°C for 24 h. The samples (0.5 g) along with operational blanks and standard solution of known concentrations were digested in 5 ml of distilled nitric acid (Analytical Reagent Grade, Merck) at 140°C for 45 min in a Microwave Digestion System (Perkin Elmer) to obtain clear digests. Following digestion, the volume of each sample was made up to 25 ml using Milli-Q water and elemental determination was performed by ICP-OES. For calculating the grain micronutrient concentration, the mean of element specific blank concentration was subtracted from each data point. The data were then multiplied by initial sample volume, divided by initial weight of grains, and expressed as μg element g^-1^ dry grain material (ppm) (Khokhar et al., 2018).

### Statistical Analysis

The statistical parameters including mean, standard deviation, coefficient of variation (CV), frequency distribution, and analysis of variance (ANOVA) for the grain micronutrient concentrations were calculated in the R statistical package. The broad heritability [*H*^2^ = VG/(VG + VE)] for each trait was estimated individually by considering genetic (VG), environmental (*VE*), and error variance (VE). Variance components for all traits were analyzed using general linear model to detect the effect of genotypes and years using one-way ANOVA. Phenotypic best linear unbiased predictor (BLUP) was estimated for each accession and trait using the lme4 package in R (Bates et al., 2014) and these values were used for correlation analysis between grain size and micronutrients concentration.

### Genotyping and Marker Trait Association Analysis

*Ae. tauschii* accessions were genotyped using the genotyping-by-sequencing (GBS) method as described in Poland et al. (2012). Briefly, the raw Illumina data were trimmed to 64 bp tags and unique tags were internally aligned to find putative SNPs. The Fisher exact test was used to determine if the two alleles were independent SNP markers. The SNPs with minor allele frequency above 5% and missing data less than 70% were positioned in the Synthetic × Opata reference genome map (Chapman et al., 2015). Detailed information on SNP genotyping and population structure of these *Ae. tauschii* accessions has been described previously (Arora et al., 2017).

Genotyping-by-sequencing-based SNP markers were used to find the genetic identity between the accessions. From the group of accessions that had >99% genetic identity and high phenotypic similarity, only single accessions were retained for further marker trait association (MTA) studies. The AM was conducted for 114 non-redundant accessions using 5,249 SNP markers on the BLUP values of each phenotype. For conducting MTA, a R GWAS package called FarmCPU (Fixed and random model Circulating Probability Unification) (Liu et al., 2016) was used. It used first three components of PCA as covariate in the regression model and calculated the *p*-value threshold for each trait by using 1,000 permutations. The *p*-value distribution for four micronutrients was shown in quantile–quantile (Q-Q) plot. To search for the putative candidate genes associated with these markers, we determined the LD decay for both the lineages. The tags were mapped to the *Ae. tauschii* reference genome (Luo et al., 2017) to get their physical coordinates and LD estimates between marker pairs were obtained using TASSEL v5 for both the lineages. We took the 95th percentile of *r*^2^ values as the estimator of short-range LD, and the distance at which this short-range LD is halved as the estimator of LD distance.

## Results

### Phenotypic Variation

**FIGURE 1:**
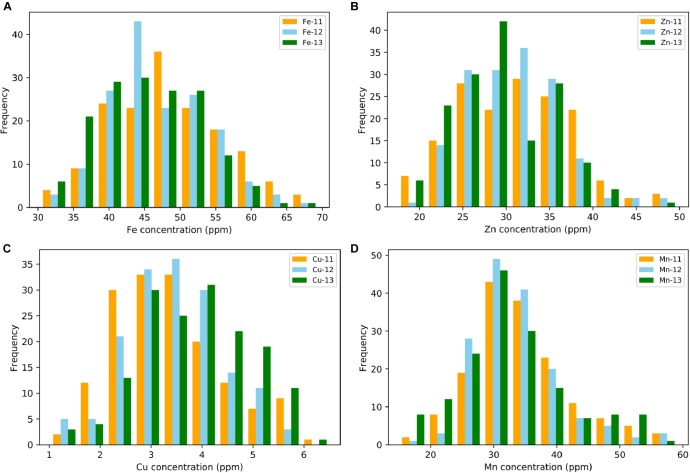
Phenotypic distribution for grain **(A)** iron—Fe, **(B)** zinc—Zn, **(C)** copper—Cu, and **(D)** manganese—Mn in year 2011, 2012, and 2013.

A wide range of variation for all four grain micronutrients was observed in the *Ae. tauschii* panel from the seed harvested in three consecutive years 2011–2013 (Figure 1). The variation ranged from 30.33 to 69.44 ppm (mean ± SD = 47.26 ± 7.58 ppm) for grain Fe, 17.54 to 49.78 ppm (30.73 ± 5.88 ppm) for grain Zn, and 1.02 to 6.50 ppm (3.62 ± 1.02 ppm) for grain Cu and 15.02 to 59.10 ppm (33.58 ± 7.7 ppm) for grain Mn concentration. A total of 2.28-, 2.83-, 6.37-, and 3.93-fold variation for Fe, Zn, Cu, and Mn, respectively, was observed among the 167 *Ae. tauschii* accessions. Grain Mn concentration had the highest heritability (0.67), while Zn had the lowest heritability (0.37). For Fe and Cu, the heritability estimates were 0.42 and 0.53, respectively (Table 1). Both Fe and Zn concentrations were slightly higher in 2011 than 2012 and 2013 whereas Cu concentration was higher for 2013 (Supplementary Figure S1). This variation can be attributed to environmental effects. ANOVA showed significant effects of the genotypes and the year on micronutrient concentration in grains. Compared with the two bread wheat cultivars used as check in the study, PBW343 and WL711, the concentration of all four micronutrients was significantly higher in the *Ae. tauschii* germplasm (Supplementary Table S1). Both these wheat lines are widely grown cultivars in India, especially PBW343 which has the 1BL/1RS translocation.

**Table 1:** Descriptive statistics, broad sense heritability (H^2^), and *F*-value from analysis of variance for the grain micronutrients concentration in year 2011, 2012, and 2013.

Micronutrient	Year	Mean ± SD (ppm)	CV%	Range		*H*^2^	*F*-values from ANOVA	
						
				Min	Max		Year	Genotype
Iron	2011	48.55 ± 8.23	16.9	31.10	69.44			
	2012	47.36 ± 6.98	14.7	30.33	65.59			
	2013	45.88 ± 7.28	15.8	30.82	69.39			
	2011–2013	47.26 ± 7.58	16.0	30.33	69.44	0.42	8.07^∗∗∗^	3.12^∗∗∗^
Zinc	2011	31.48 ± 6.21	19.7	17.54	47.06			
	2012	30.85 ± 5.55	17.9	19.90	49.78			
	2013	29.86 ± 5.81	19.4	18.14	46.68			
	2011–2013	30.73 ± 5.88	19.1	17.54	49.78	0.37	4.87^∗∗^	3.03^∗∗∗^
Copper	2011	3.38 ± 1.05	30.3	1.02	6.50			
	2012	3.46 ± 0.91	26.0	1.35	5.88			
	2013	3.82 ± 1.05	26.9	1.20	6.27			
	2011–2013	3.62 ± 1.02	28.4	1.02	6.50	0.53	18.6^∗∗∗^	4.14^∗∗∗^
Manganese	2011	34.42 ± 7.95	24.6	16.07	59.10			
	2012	33.41 ± 6.83	21.0	16.25	57.62			
	2013	32.91 ± 8.53	27.0	15.02	55.28			
	2011–2013	33.58 ± 7.7	24.3	15.02	59.10	0.67	4.13^∗^	7.71^∗∗∗^


*ns: not significant; ^∗∗∗^, ^∗∗^, and ^∗^, significant at *P* < 0.001, *P* < 0.01, and *P* < 0.05, respectively.*

### Relationship Between Grain Micronutrients and Grain Size

**FIGURE 2:**
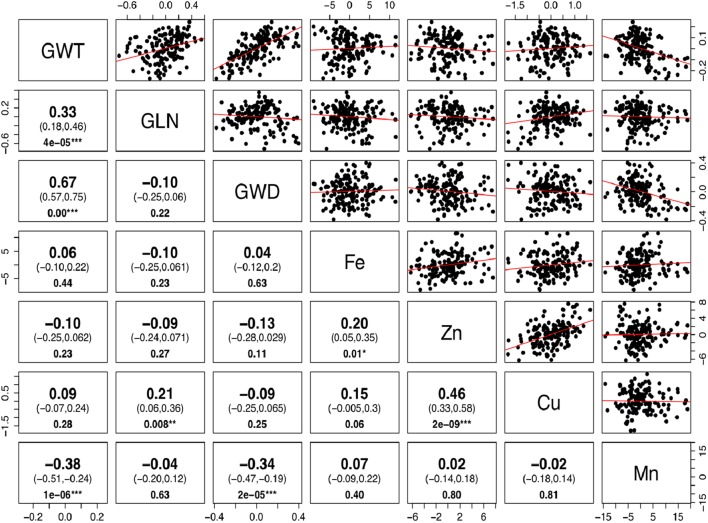
Correlation for grain size and micronutrients concentration in *Ae. tauschii* accessions. Phenotypic correlations between 50-grain weight (GWT), grain length (GLN), grain width (GWD), iron (Fe), zinc (Zn), copper (Cu), and manganese (Mn) concentrations. The upper and lower 95% confidence intervals are included in parenthesis below the correlation value. *P*-value for significant correlations is shown at the bottom. (Note: ^∗∗∗^, significant at *P* < 0.001; ^∗∗^, significant at *P* < 0.01; ^∗^, significant at *P* < 0.05.)

The phenotypic values for the 3 years were converted into BLUP values to get unbiased mean estimates. A strong positive linear relationship was found between BLUPs and mean values with the shrinkage of BLUPs toward the population average. The BLUP values depicted a normal distribution for grain Fe, Zn, and Cu concentration (Supplementary Figure S2). Significant positive correlations between grain Fe, Zn, and Cu concentrations were observed, while Mn did not show any significant correlation with other minerals (Figure 2). As micronutrient concentrations are highly influenced by the environment, correlations of the four micronutrients were also assessed across years. A strong positive correlation was observed between years for grain Mn concentration (*r* = 0.65–0.70), whereas for grain Cu (*r* = 0.49–0.56), Fe (*r* = 0.37–0.45), and Zn (*r* = 0.26–0.44), moderate positive correlations were found between the years (Supplementary Figure S3). The high correlation observed for Mn between the years also explains its high heritability value compared to other micronutrients.

There is a perception that higher micronutrient concentration in wild species is a result of concentration effects due to smaller seeds. To determine whether seed size has any significant effect on micronutrient concentrations, we estimated correlations between the BLUP values for grain micronutrient concentrations and grain weight of these accessions. Very weak to almost no correlation was observed between grain weight and grain Fe, Zn, and Cu concentrations with Pearson correlation coefficient of 0.06, -0.10, and 0.09, respectively. Grain Mn concentration, however, had significant but negative correlation with grain weight (Figure 2). Detailed dissection of grain architecture has been reported in Arora et al. (2017).

### Variation Between Lineages

**FIGURE 3:**
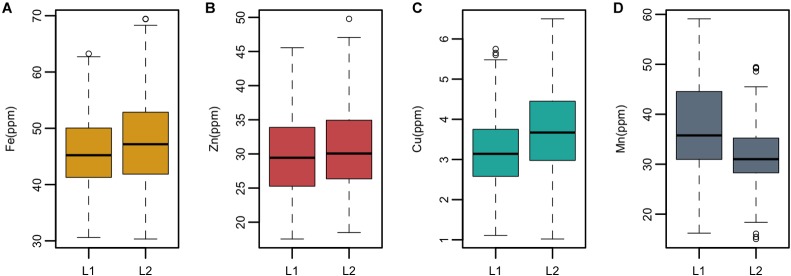
Boxplots showing mean, median, and range of phenotypic variation between the two lineages L1 and L2 of *Ae. tauschii* for grain **(A)** iron—Fe, **(B)** zinc—Zn **(C)**, copper—Cu, and **(D)** manganese—Mn.

*Ae. tauschii* is genetically divided into two lineages which are referred to as L1 and lineage 2 (L2). L1 predominantly encompasses accessions belonging to subspecies *tauschii* and L2 accessions belonging to ssp. *strangulata*. Significant differences (*p* > 0.05) in the mean values of grain Fe, Cu, and Mn concentrations were detected between the two lineages; however, no significant difference was observed for zinc concentration (Supplementary Table S2). Overall, L2 had significantly higher concentrations of grain Fe and Cu than L1, whereas L1 had higher Mn concentration than L2 as depicted by box plots in Figure 3.

In this study, we have identified several accessions with high mean value for Fe, Zn, and Cu concentrations. Three accessions pauT14334, pauAT14145, and pauAT3751 had high iron concentration with an average of 63.78, 63.67, and 62.58 ppm, respectively. Four accessions pauAT14360, pauAT14136, pauAT14158, and pauAT14139 accumulated the highest concentration for both iron and zinc with an average of 56.73, 53.25, 55.28, and 56.13 ppm iron and 41.70, 40.79, 38.74, and 39.80 ppm zinc, respectively. Accession pauAT14162 was found to have high concentrations of both Zn and Cu with mean values of 46.45 and 5.25 ppm, respectively. The accessions reported for higher Mn concentration were moderate for other micronutrients. Most accessions with higher concentration of Fe, Zn, and Cu belonged to L2; however, accessions with higher Mn concentration belonged to L1 (Table 2). This was also observed when the two lineages were compared for all micronutrients (Figure 3).

**Table 2:** List of selected accessions of *Ae. tauschii* with high grain iron (Fe), zinc (Zn), copper (Cu), and manganese (Mn) concentrations.

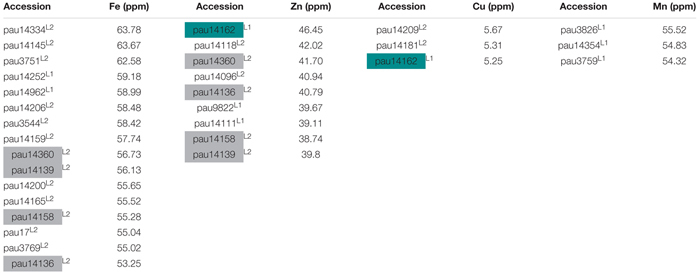

*The lineage is mentioned for each accession as superscript. Accessions with both high Fe and Zn are colored gray and those with high Zn and Cu are colored turquoise green.*

### Detection of Marker Trait Associations

**FIGURE 4:**
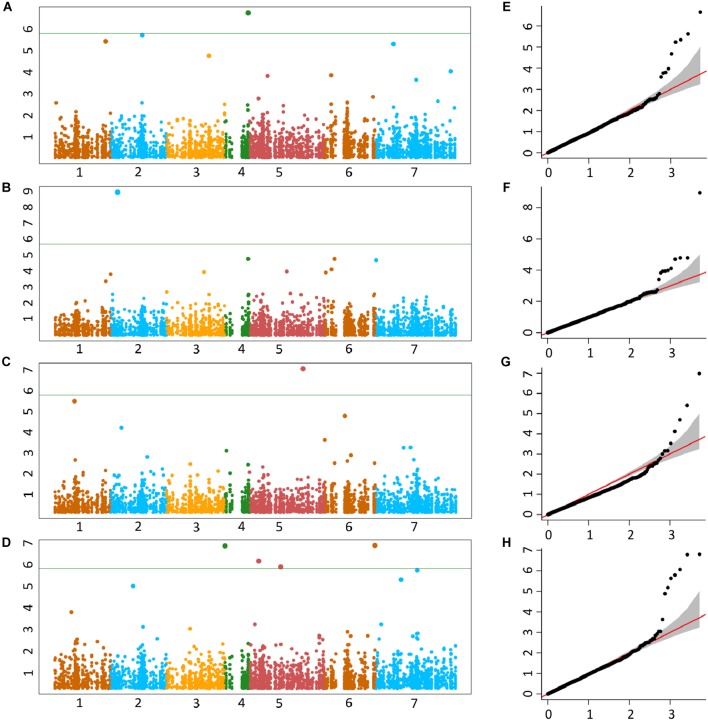
Manhattan plots representing seven chromosomes carrying the significant markers detected by MLM models using BLUP values for grain **(A)** Fe, **(B)** Zn, **(C)** Cu, and **(D)** Mn. Quantile–quantile (Q-Q) plots for grain Fe, Zn, Cu, and Mn **(E–H)** showing expected null distribution of *p*-values, assuming no associations, represented as red solid line; distribution of *p-*values observed using mixed linear model (MLM) represented as a black dots.

The genetic basis of accumulation of Fe, Zn, Cu, and Mn in the grains of *Ae. tauschii* was studied using genome wide AM. GWAS analysis was performed using FarmCPU for 114 non-redundant *Ae. tauschii* accessions with 5,249 SNP markers. Population structure for this panel was inferred by principal component analysis (PCA) in our previous study (Arora et al., 2017). The accessions were divided into two major clusters, L1 and L2, with some intermediate accessions represented as admixture (Supplementary Figure S4). The FarmCPU used first three components of PCA as covariate in association analysis. In FarmCPU, the default *p*-value threshold is the Bonferroni-corrected threshold (indicated by the green line in Manhattan plots). As the Bonferroni-corrected threshold is overly strict, it allows to calculate threshold using the “p.threshold” function which permutes the phenotypes to break the relationship with the genotypes. We permuted the phenotypes 1,000 times, a vector of minimum p value of each experiment was outputted and the 95% quantile value of the vector was used as p.threshold in this study. This method gave -log(*p*-value) of 4.68 which was used as a cut-off to define significant associations. There were 19 MTAs above the threshold -log(P) score of 4.68, distributed on all the seven *Ae. tauschii* chromosomes. The details of these MTAs are summarized in Table 3 and depicted as Manhattan plots in Figure 4A–D. The Q-Q plots illustrating observed associations between SNPs and grain micronutrient concentrations compared to expected associations after accounting for population structure are presented in Figure 4E–H.

**Table 3:** List of significant marker loci associated with BLUP values of grain micronutrient (Fe, Z, Cu, Mn) concentration.

Trait	SNP ID	Chromosome	Position ^#^(cM)	*p*-value	MAF	Effect^∗^	-log(*p*-value)
Fe	AT68157	4D	66.6	2.16E-07	0.20	3.45	6.67
	AT76904	2D	89.9	2.35E-06	0.23	2.38	5.63
	AT45556	1D	143.5	4.45E-06	0.22	-2.98	5.35
	AT2276	7D	51.6	5.80E-06	0.40	4.03	5.24
	AT88633	3D	120.8	2.07E-05	0.26	1.47	4.68
Zn	AT2707	2D	19.7	1.08E-09	0.21	3.39	8.97
	AT65894	4D	65.5	1.61E-05	0.12	2.80	4.79
	AT77346	6D	29.8	1.63E-05	0.18	-2.16	4.79
	AT92754	7D	1.1	1.98E-05	0.33	2.59	4.70
Cu	AT75576	5D	151.8	1.03E-07	0.28	-0.76	6.99
	AT62347	1D	55.9	3.86E-06	0.07	0.49	5.41
	AT37896	6D	58.6	2.04E-05	0.21	0.31	4.69
Mn	AT105092	6D	144.0	1.55E-07	0.49	-5.74	6.81
	AT102954	4D	1.0	1.61E-07	0.19	-5.96	6.79
	AT33443	5D	27.6	8.56E-07	0.23	2.39	6.07
	AT359	5D	89.6	1.60E-06	0.14	2.68	5.80
	AT78733	7D	117.5	2.29E-06	0.11	3.16	5.64
	AT4038	7D	71.6	6.51E-06	0.41	2.17	5.19
	AT102015	2D	64.6	1.31E-05	0.11	-2.34	4.88


*MAF, minor allele frequency.*

*^#^Position of the SNPs is according to POPSEQ data (Chapman et al., 2015; Edae et al., 2015).*

*^∗^An allelic effect size is the magnitude of the effect of an allele on a phenotype. Sign of the allelic effect estimate is with respect to the nucleotide that is second in alphabetical order. For associated SNP markers, where positive effects have been reported second allele in alphabetical order is favorable, and where negative effects have been reported, the first allele is favorable. This is illustrated by allele specific boxplots in Figure 6.*

**FIGURE 5:**
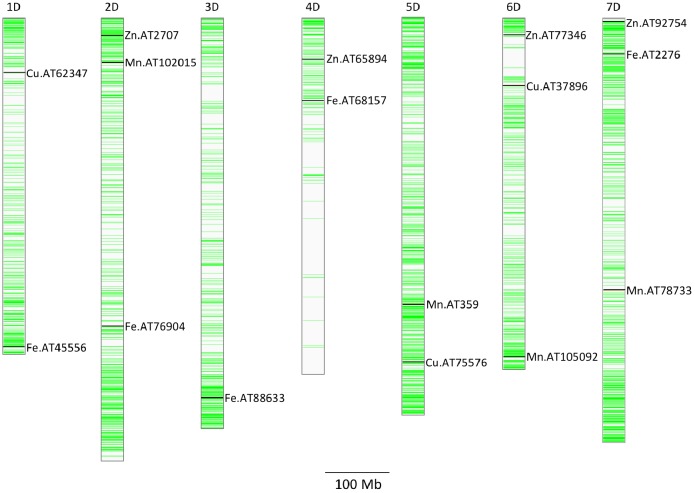
Distribution of GBS SNP markers across the seven *Ae. tauschii* chromosome arms. The horizontal line color bars indicate the chromosomal position of the markers. The associated markers for micronutrients concentration reported in this study are indicated by black bars. Associated SNPs are indicated by name of the micronutrient followed by AT indicating *Ae. tauschii* and SNP number.

**FIGURE 6:**
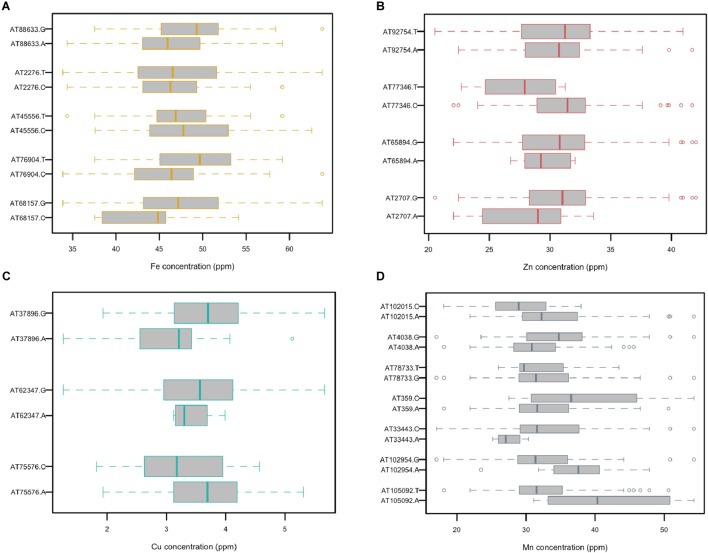
Comparison of the allelic effects for the SNP markers associated with grain **(A)** Fe, **(B)** Zn, **(C)** Cu, and **(D)** Mn concentration.

A total of five, four, three, and seven MTAs were detected for Fe, Zn, Cu, and Mn, respectively, with log(*p*-value) of ≥4.68. For Fe, the most significant MTA was detected on chromosome 4D followed by chromosomes 2D, 1D, 7D, and 3D (Figure 5). For Zn, the significant MTAs were detected on chromosomes 2D, 4D, 6D, and 7D. Fe and Zn MTAs on 4D were located in the mapping bins 1.1 cM apart. MTAs for Cu were found on chromosomes 5D, 1D, and 6D while Mn MTAs were mapped on chromosomes 6D, 4D, 5D, 7D, and 2D. The allelic effects of the significant linked SNP markers were determined by calculating mean grain micronutrient concentrations for both the SNP alleles individually and represented as box plots in Figure 6.

### Candidate Genes

To define the search space for putative candidate genes in the vicinity of associated markers, the LD decay distance was determined for both the lineages. The decay for L1 and L2 was at 98 and 177 kb, respectively (Supplementary Figure S5). We first used the Wheat reference Chinese Spring (CS) RefSeq v1.0 genome (International Wheat Genome Sequence Consortium, 2018) to search for genes, since it has been well-annotated compared to *Ae. tauschii* reference, and took the L2 LD block (177 kb) to define the gene search space around the marker because of the proximity of CS D-genome to the L2 (Wang et al., 2013). We mapped the SNP markers to CS RefSeq v1.0 genome (Ref) and fetched the annotated genes in 177-kb region around the marker^1^. For these candidates, we looked for gene networks on KnetMiner^2^ and reported the candidates that were associated with micronutrient accumulation (Table 4).

**Table 4:** Candidate gene predicted in genomic regions harboring grain micronutrient marker trait associations.

SNP ID	Candidate genes	Function
Fe/AT45556	Putative ADP-ribosylation factor	Vesicle transport
Fe/AT2276	AT-hook motif nuclear-localized protein	DNA binding motif
	FAD/NAD(P)-binding domain	Oxidation–reduction process
	Kinesin motor domain	Microtubule motor activity; organelle transport
	YEATS	Regulation of transcription
Fe/AT_68157	Pentatricopeptide repeat	RNA-binding proteins
	Glycosyl transferase, family 1	Acetylglucosaminyltransferase activity
	Response regulator receiver domain	Signal transduction response regulator
Fe/AT76904	FAD/NAD(P)-binding domain	Oxidation–reduction process
	Cytochrome oxidase assembly protein 1	Mitochondrial membrane protein
	WRKY domain	Transcription factor activity
	VHS and GAT domain	Vesicular trafficking
Zn/AT65894	HVA22-like protein with RNA recognition motif	Development
	WD40/YVTN repeat-like-containing domain	Protein binding
	NAC domain	Regulation of transcription
Zn/AT2707	Scarecrow-like 3 (SCL3)	GRAS transcription regulator
	UDP-glucuronosyl/UDP-glucosyltransferase	Regulation of ion transmembrane
	ABC transporter	ATPase activity coupled with transmembrane movement of substances
Zn/AT77346	Malonyl-coenzyme A: anthocyanin 3-*O*-glucoside-6″-*O*-malonyltransferase	Development
	Ribosome-inactivating protein	rRNA N-glycosylase activity
	Catalase immune-responsive domain (CAT3)	Catalase activity
	Bifunctional inhibitor	Plant lipid transfer protein
Zn/AT92754	Zinc-binding in reverse transcriptase with zf-RVT domain	Not known
	Zinc finger, PMZ-type	Zinc ion binding
	Kelch-type beta propeller	Protein binding
	NB-ARC and LRR	ADP binding
Cu/AT75576	FAD/NAD(P)-binding domain; GDP dissociation inhibitor; Guanylate-binding protein	Oxidoreductase activity; protein transport
	BTB/POZ domain; MATH/TRAF domain	Protein binding
	Ulp1 protease family, C-terminal catalytic domain	Cysteine-type peptidase activity
Cu/AT62347	Reverse transcriptase zinc-binding domain	Zinc-binding in reverse transcriptase
Cu/AT37896	F-box domain	Protein binding
	EF-hand binding site	Protein binding
Mn/AT105092	TCP21-like	Transcription factor
	F-box domain; Phloem protein 2-like	Protein binding
Mn/AT359	Auxin-responsive protein AtMHX	Metal homeostasis
Mn/AT102015	F-box domain containing protein	Protein binding
	NADH-ubiquinone reductase complex 1	ATP generation
	K+ potassium transporter	Potassium ion transmembrane transporter activity
Mn/AT102954	Serine-threonine/tyrosine-protein kinase	Protein kinase activity
Mn/AT33443	Kinesin motor domain	ATP binding; microtubule motor activity
Mn/AT78733	Glycosyl transferase	Acetylglucosaminyltransferase activity


Based on these search criteria, we found some candidate genes in the vicinity of these markers that were associated with vesicle transport, development, and transcription regulation. The Fe MTA AT45556 on chromosome 1D mapped in close proximity of the gene ADP- ribosylation factor (ARF), important in vesicle transport and involved in the diurnal changes in mugineic acid family phytosiderophores (MAs) secretion (Nozoye et al., 2004; D’Souza-Schorey and Chavrier, 2006). Another candidate gene for Fe concentration underlying marker AT2276 on chromosome 7D encodes an AT-hook motif nuclear localized protein which functions in the regulation of gene expression.

The Zn MTA AT65984 on chromosome 4D mapped adjacent to an abscisic acid-induced protein, HVA22 which inhibits gibberellin (GA)-mediated programmed cell death in cereal aleurone cells and acts as a positive factor for metal accumulation under stress conditions (Singh et al., 2014). AT2707, associated with Zn concentration on chromosome 2D, lies close to the predicted Scarecrow-like 3 (SCL3) GRAS transcription regulator. It functions as a positive regulator to integrate and maintain a functional GA pathway (Zhang et al., 2011). An interesting candidate gene called ABC transporter is also associated with AT2707, and is involved in the export or import of a wide variety of substrates ranging from small ions to macromolecules. Another Zn MTA (AT77346 on 6D) showed association with a Malonyl-coenzyme A: anthocyanin 3-*O*-glucoside-6″-*O*-malonyltransferase gene.

Cu MTA AT75576 on 5D mapped to a guanylate-binding protein which has a critical role in the regulation of a range of cellular processes including growth, differentiation, and intracellular transportation. The region around marker AT105092 on 6D for Mn was associated with a gene coding for TCP transcription factors. It constitutes a plant-specific family of developmental regulators and shares a conserved region that is predicted to form a non-canonical basic helix-loop-helix DNA-binding domain called the TCP domain (Cubas et al., 1999). 5D marker AT359 associated with grain Mn showed sequence similarity to auxin-responsive protein AtMHX, which regulates metal homeostasis mainly in tissues with photosynthetic potential (David-Assael et al., 2006). Mn marker AT102015 on 2D mapped to a gene coding for F-box domain containing protein. The extensive list of all the candidate genes associated with markers is provided in Table 4 and further investigation is required to understand the role of these candidate genes in grain micronutrients concentration.

## Discussion

Micronutrient malnutrition affects more than 2 billion people in the world, with Fe and Zn among the essential minerals that are often lacking in human diets (White and Broadley, 2009). Fe is important for oxygen transportation and hemoglobin formation, whereas Zn plays a central role in growth, development, and in the immune system (Roohani et al., 2013; Abbaspour et al., 2014). WHO data estimate that Fe-deficiency anemia in children and adults results in 19.7 million DALYs (disability-adjusted life years), or 1.3% of global total DALYs (World Health Organiztion, 2009). Therefore, increasing Fe and Zn in human diets, especially in developing countries which rely almost exclusively on cereal based diets, assumes tremendous significance.

Many studies have reported that there is a wide variation in grain Fe and Zn concentrations in wheat wild relatives. These levels of variation are significantly higher than those observed in elite wheat cultivars (Cakmak et al., 2000; Monasterio and Graham, 2000). The present investigation focused on elucidating the variation of four micronutrients in *Ae. tauschii*, the D genome donor of bread wheat. Free recombination between *Ae. tauschii* and D-genome chromosomes of bread wheat and the availability of its genome sequence makes it an attractive resource for wheat biofortification.

### *Ae. tauschii*: Potential Source for Wheat Grain Micronutrients Enrichment

In the *Ae. tauschii* accessions, almost twofold genetic variation was observed for Fe and 2.8-fold for Zn followed by Mn (3.9-fold) and Cu (6.3-fold). Even the mean concentrations of Fe, Zn, and Cu in *Ae. tauschii* panel were 1.84, 1.43, and 1.72 times to that observed in the bread wheat checks planted and analyzed along with this germplasm set. In different studies, the concentrations of Fe and Zn in elite cultivars have been reported to vary between 25–56 and 13.5–39 mg/kg, respectively (Morgounov et al., 2007; Zhao et al., 2009). In contrast, in the *Ae. tauschii* accessions studied here, Fe was as high as 69 mg/kg and Zn up to 50 mg/kg. CIMMYT and Harvest Plus have used *Ae. tauschii* for developing synthetic hexaploid wheat which were found to have better accumulation of Fe and Zn in grains than *T. aestivum* (Calderini and Ortiz-Monasterio, 2003). These studies support our assertion that the D-genome is a promising source of high micronutrient concentrations. The accessions reported in this study with higher Fe and Zn can serve as a useful source for developing synthetic hexaploid wheat.

Grain micronutrient concentrations are quantitatively inherited traits, as shown by the continuous distribution. Genetic variance was low to moderate (range, 0.16–0.58), indicating high environmental influence on trait expression and/or complex genetic architecture. High genotype × environment interactions for grain nutrient concentrations have been reported for both wheat and wild emmer wheat (Oury et al., 2006; Morgounov et al., 2007; Chatzav et al., 2010). These studies suggested that genotype × environment interactions are non-cross over interactions, and therefore reasonable advances in selection and breeding can be expected.

*Ae. tauschii* is genetically divided into two diverse lineages which are referred to as L1 and L2. L1 consists of subsp. *tauschii var. typica* and *anathera* and L2 consists of subsp. *stangulata* and subsp. *tauschii var. meyeri* (Kihara and Tanaka, 1958). A very conspicuous observation was the differential accumulation of micronutrients in the two *Ae. tauschii* subspecies. The Student’s *t*-test revealed significant (*p* > 0.05) difference in the mean values of grain Fe, Cu, and Mn concentrations between the two lineages; however, no significant difference was observed for Zn concentration. L2 accessions had the highest mean values for all traits studied except grain Mn which was highest in L1. Mansouri et al. (2013) evaluated 15 morphological characters of *Ae. tauschii* and concluded that ssp. *strangulata* has higher mean values for most of the traits including 100-grain weight. Research conducted by Chatzav et al. (2010) in wild emmer found significant differences in grain nutrient concentrations between the two groups (northern and southern), albeit of negligible magnitude. To our knowledge, these findings are the first study to report significant micronutrient differences between the two lineages of *Ae. tauschii*. So far, numerous genetic studies were conducted based on molecular markers to differentiate these lineages.

A significantly positive relationship was observed between grain Fe and Zn concentration (*r*^2^ = 0.20) and Zn–Cu concentration (*r*^2^ = 0.46). The positive correlation suggests that there could be common genetic factors affecting the accumulation of these micronutrients in grains. The existence of positive correlations between grain iron and zinc has been reported repeatedly in bread wheat (Zhao et al., 2009; Xu et al., 2012; Srinivasa et al., 2014), wild emmer (Cakmak et al., 2004; Peleg et al., 2008), and triticale (Feil and Fossati, 1995). However, the co-localization of QTL for grain Fe and Zn has been reported in tetraploid wheat (Uauy et al., 2006; Peleg et al., 2009).

Correlation coefficients between grain weight and Fe, Zn, and Cu concentrations were very low (*r* = 0.06, -0.10, and 0.09, respectively) and non-significant. The hypothesis that grain weight may affect grain micronutrients concentration was not supported by the data in this study, as no significant correlations were observed between grain size and Fe, Zn, and Cu accumulation. Identification of some of the *Ae. tauschii* accessions with larger seeds and higher micronutrient concentration (pau14360, pau14159, pau14139, pau14158, pau14334, pau14136) contradict this concept that higher micronutrient concentration in wild species is a result of concentration effect due to smaller seeds. Similar results showing no concentration effect in wild species were reported in A-genome diploid wheat (Tiwari et al., 2009), durum wheat (Ficco et al., 2009), *T. dicoccoides* accessions (Cakmak et al., 2004), and wheat cultivars (Morgounov et al., 2007). On the other side, Mn concentration was significantly and negatively correlated with grain weight and width (*r* = 0.38, *r* = 0.34, respectively). An interesting finding of this study is that L1 was found to have accessions with smaller grain size and higher Mn concentration. Of the four micronutrients studied, only Mn accumulation in grains was affected by smaller grain size (concentration effect).

Various studies had been conducted in wheat to map the QTLs responsible for Fe and Zn concentration. GWAS in *Ae. tauschii* identified QTL for grain micronutrient concentrations on all seven chromosomes with each chromosome harboring QTL for more than one micronutrient. Tiwari et al. (2009) mapped QTL for grain Fe and Zn concentration in a RIL population of diploid A genome wheat *T. monococcum* and *T. boeoticum*. The significant QTLs for grain Fe were located on chromosomes 2A and 7A and for Zn on chromosome 7A. *Ae. tauschii* chromosomes 2D and 7D also located one QTL each for grain Fe, Zn, and Mn though the locations of these QTL were different indicating independent genetic elements controlling these three traits. Another work by Shi et al. (2008) detected as many as four QTLs for grain Zn concentration and seven for grain Zn content. The QTL detected on chromosome 7A explained the highest level of phenotypic variation. Chromosome 5D and 6D did not map any loci for grain Fe in the present study and 1D did not have any association with grain Zn and Mn.

Annotation of the 177-kb genomic regions in CS genome on either side of the SNPs associated with micronutrient grain content identified some genes hypothesized to be directly involved in micronutrient acquisition and translocation. The release of phytosiderophores (PSs) by grass species is considered a highly efficient Fe acquisition mechanism. These low-molecular-weight, nonproteinogenic amino acids form soluble complexes with Fe(III) that are taken up as the intact PS–metal complex, with Fe remaining in its oxidized form, Fe(III) (Oburger et al., 2014). MTA AT45556 annotated a putative ADP-ribosylation factor involved in vesicle transport and has been reported to contribute to diurnal changes in the expression of genes that participate in PS synthesis in rice (Nozoye et al., 2004). ADP-ribosylation factor might also be regulating synthesis of phytosedirophores from *Ae. tauschii* roots. *Aegilops* species have been reported to release two to four times higher PS than cultivated wheats (Kumari et al., 2010). The associated markers Mn/AT102015 and Zn/AT65984 were mapped close to the genes coding for abscisic acid-induced protein, HVA22, and F-box domain containing protein, respectively. Both these proteins were found to exhibit upregulation of transcripts in grains of the high mineral wheat variety compared to a low mineral variety. The proteins increased tolerance to stress during grain filling, which was suggested as a positive factor for metal accumulation (Singh et al., 2014). The SNP marker AT359 associated with auxin-responsive protein AtMHX is an auxin regulated vacuolar transporter functions in metal homeostasis. It exchanges protons with Mg2+, Zn2+, and Fe2+ ions mainly in tissues with photosynthetic potential (David-Assael et al., 2006). However, further functional validation of these genes and their role in micronutrient uptake in *Ae. tauschii* grains is still needed.

## Conclusion

To our knowledge, this is the first study to report GWAS for Fe, Zn, Cu, and Mn concentration in *Ae. tauschii* and further genetic and functional analysis of the associated genomic regions may shed light on the impact of these loci for improving micronutrient concentration of wheat. Overall, a number of accessions with high level of grain micronutrients have been identified especially for Fe and Zn which play an important role in tackling micronutrient deficiencies or hidden hunger. Bio-enriched *Ae. tauschii* accessions and genomic regions harboring grain Fe/Zn QTL provide a jumping board for developing biofortified wheat varieties.

## Author Contributions

SA carried out the phenotyping of the germplasm, analyzed both genotype and phenotype data, and wrote the draft of the manuscript. JC helped in LD analysis. CU contributed to the genome-wide association mapping, manuscript preparation, and candidate gene search. JP supervised genotyping by sequencing of the *Ae. tauschii* mapping panel, diversity analysis, and genome-wide association mapping. PC conceived the idea, designed and supervised the study, prepared the draft of the manuscript, and submitted it. All the authors have read and approved the manuscript.

## Conflict of Interest Statement

The authors declare that the research was conducted in the absence of any commercial or financial relationships that could be construed as a potential conflict of interest. The handling Editor declared a past co-authorship with the authors CU and JP.

## Footnotes

1 https://urgi.versailles.inra.fr/WheatMine/genomicRegionSearch.do

2 http://knetminer.rothamsted.ac.uk

## Supplementary Material

Figure S1 Boxplots representing variation in *Ae. tauschii* grain micronutrient concentrations for the three crop seasons—2011, 2012, 2013. The variation for grain **(A)** iron, **(B)** zinc, **(C)** copper, and **(D)** manganese concentrations observed between the years.

Figure S2 Distribution of best linear unbiased predictions (BLUP) values with normality curve for grain **(A)** iron, **(B)** zinc, **(C)** copper, and **(D)** manganese.

Figure S3 Correlation matrix for grain micronutrient concentrations in *Ae. tauschii* for year 2011–2013. The values in the column are correlation coefficient at the top and *p*-value for significant correlations is shown at the bottom. The symbols ^∗∗∗^, ^∗∗^, and ^∗^, indicate significant at *p* < 0.001, *p* < 0.01, and *p* < 0.05, respectively. The colors represent strength of correlation from strongly positive (strong red) to negative (regent blue).

Figure S4 The heat map for non-redundant *Ae. tauschii* accessions created using GAPIT. The panel is genetically divided into two diverse lineages which are referred to as lineage 1 (L1) and lineage 2 (L1).

Figure S5 The LD decay plot for lineage 1 and 2 of *Ae. tauschii*.

Table S1 Comparison of micronutrient concentration between hexaploid wheat cultivars (WL711 and PBW343) and *Aegilops tauschii* germplasm.

Table S2 Descriptive statistics of *Ae. tauschii* accessions for micronutrients concentration based on two lineages (L1, L2).

## References

[B1] Abbaspour N. Hurrell R. Kelishadi R. (2014). Review on iron and its importance for human health. *J. Res. Med. Sci.* 19 164–174. 24778671 PMC3999603

[B2] Arora S. Singh N. Kaur S. Bains N. S. Uauy C. Poland J. (2017). Genome-wide association study of grain architecture in wild wheat *Aegilops tauschii*. *Front. Plant Sci.* 8:886. 10.3389/fpls.2017.00886 28620398 PMC5450224

[B3] Bates D. Ozkan H. Braun H. J. Welch R. M. Romheld V. (2014). *Fitting Linear Mixed-Effects Models Using Lme4.* Available at: https://arxiv.org/pdf/1406.5823.pdf

[B4] Bouis H. E. Welch R. M. (2010). Biofortification—a sustainable agricultural strategy for reducing micronutrient malnutrition in the global south. *Crop Sci.* 50 S–20–S–32 10.2135/cropsci2009.09.0531

[B5] Cakmak I. Feldman M. Fahima T. Korol A. Nevo E. Braun H. J. (2004). *Triticum dicoccoides*: an important genetic resource for increasing zinc and iron concentration in modern cultivated wheat. *Soil Sci. Plant Nutr.* 50 1047–1054. 10.1080/00380768.2004.10408573

[B6] Cakmak I. Ozkan H. Braun H. J. Welch R. M. Romheld V. (2000). Zinc and iron concentrations in seeds of wild, primitive, and modern wheats. *Food Nutr. Bull.* 21 401–403. 10.1177/156482650002100411

[B7] Calderini D. F. Ortiz-Monasterio I. (2003). Are synthetic hexaploids a means of increasing grain element concentrations in wheat? *Euphytica* 134 169–178. 10.1023/B:EUPH.0000003849.10595.ac

[B8] Chapman J. A. Mascher M. Buluç A. Barry K. Georganas E. Session A. (2015). A whole-genome shotgun approach for assembling and anchoring the hexaploid bread wheat genome. *Genome Biol.* 16:26. 10.1186/s13059-015-0582-8 25637298 PMC4373400

[B9] Chatzav M. Peleg Z. Ozturk L. Yazici A. Fahima T. Cakmak I. (2010). Genetic diversity for grain nutrients in wild emmer wheat: potential for wheat improvement. *Ann. Bot.* 105 1211–1220. 10.1093/aob/mcq024 20202969 PMC2887062

[B10] Chhuneja P. Dhaliwal H. S. Bains N. S. Singh K. (2006). Aegilops kotschyi and *Aegilops tauschii* as sources for higher levels of grain Iron and Zinc. *Plant Breed.* 125 529–531. 10.1111/j.1439-0523.2006.01223.x

[B11] Cubas P. Lauter N. Doebley J. Coen E. (1999). The TCP domain: a motif found in proteins regulating plant growth and development. *Plant J.* 18 215–222. 10.1046/j.1365-313X.1999.00444.x 10363373

[B12] David-Assael O. Saul H. Saul V. Mizrachy-Dagri T. Berezin I. Brook E. (2006). AtMHX is an auxin and ABA-regulated transporter whose expression pattern suggests a role in metal homeostasis in tissues with photosynthetic potential. *Funct. Plant Biol.* 33:661 10.1071/FP05295 32689275

[B13] D’Souza-Schorey C. Chavrier P. (2006). ARF proteins: roles in membrane traffic and beyond. *Nat. Rev. Mol. Cell Biol.* 7 347–358. 10.1038/nrm1910 16633337

[B14] Edae E. A. Bowden R. L. Poland J. (2015). Application of population sequencing (POPSEQ) for ordering and imputing genotyping-by-sequencing markers in hexaploid wheat. *G3 (Bethesda)* 5 2547–2553. 10.1534/g3.115.020362 26530417 PMC4683627

[B15] Feil B. Fossati D. (1995). Mineral composition of triticale grains as related to grain yield and grain protein. *Crop Sci.* 35:1426 10.2135/cropsci1995.0011183X003500050028x

[B16] Ficco D. B. M. Riefolo C. Nicastro G. De Simone V. Gesù A. M. Beleggia R. (2009). Phytate and mineral elements concentration in a collection of Italian durum wheat cultivars. *Field Crops Res.* 111 235–242. 10.1016/J.FCR.2008.12.010

[B17] International Wheat Genome Sequence Consortium (2018). Shifting the limits in wheat research and breeding through a fully annotated and anchored reference genome sequence. *Science* 361:eaar7191 10.1126/science 30115783

[B18] Khokhar J. S. Sareen S. Tyagi B. S. Singh G. Wilson L. King I. P. (2018). Variation in grain Zn concentration, and the grain ionome, in field-grown Indian wheat. *PLoS One* 13:e0192026. 10.1371/journal.pone.0192026 29381740 PMC5790267

[B19] Khush Sichul G. S. Lee S. Cho J. I Jeon J. S. (2012). Biofortification of crops for reducing malnutrition. *Plant Biotechnol. Rep.* 6 195–202. 10.1007/s11816-012-0216-5

[B20] Kihara H. Tanaka M. (1958). *Morphological and Physiological Variation Among Aegilops Squarrosa Strains Collected in Pakistan, Afghanistan and Iran.* Available at: http://www.sidalc.net/cgi-bin/wxis.exe/?IsisScript=UACHBC.xis&method=post&formato=2&cantidad=1&expresion=mfn=032677

[B21] Kumari N. Tiwari V. K. Rawat N. Tripathi S. K. Randhawa G. S. Dhaliwal H. S. (2010). Identification of Aegilops species with higher production of phytosiderophore and iron and zinc uptake under micronutrient-sufficient and -deficient conditions. *Plant Genet. Resourc.* 8 132–141. 10.1017/S1479262110000080

[B22] Liu X. Huang M. Fan B. Buckler E. S. Zhang Z. (2016). Iterative usage of fixed and random effect models for powerful and efficient genome-wide association studies. *PLoS Genet.* 12:e1005767. 10.1371/journal.pgen.1005767 26828793 PMC4734661

[B23] Luo M.-C. Gu Y. Q. Puiu D. Wang H. Twardziok S. O. Deal K. R. (2017). Genome sequence of the progenitor of the wheat D genome *Aegilops tauschii*. *Nature* 551 498–502. 10.1038/nature24486 29143815 PMC7416625

[B24] Mansouri S. Mehrabi A. A. Kahrizi D. (2013). Evaluation of genetic diversity of *Aegilops tauschii* accessions using morphological characters. *J. Crop Sci. Biotechnol.* 16 197–200. 10.1007/s12892-013-0017-6 19093488

[B25] Monasterio I. Graham R. D. (2000). *Breeding for Trace Minerals in Wheat.* Available at: http://journals.sagepub.com/doi/pdf/10.1177/156482650002100409 10.1177/156482650002100409

[B26] Morgounov A. Gómez-Becerra H. F. Abugalieva A. Dzhunusova M. Yessimbekova M. Muminjanov H. (2007). Iron and zinc grain density in common wheat grown in Central Asia. *Euphytica* 155 193–203. 10.1007/s10681-006-9321-2

[B27] Nozoye T. Itai R. N. Nagasaka S. Takahashi M. Nakanishi H. Mori S. (2004). Diurnal changes in the expression of genes that participate im phytosiderophore synthesis in rice. *Soil Sci. Plant Nutr.* 50 1125–1131. 10.1080/00380768.2004.10408585

[B28] Oburger E. Gruber B. Schindlegger Y. Schenkeveld W. D. Hann S. Kraemer S. M. (2014). Root exudation of phytosiderophores from soil-grown wheat. *New Phytol.* 203 1161–1174. 10.1111/nph.12868 24890330 PMC4143957

[B29] Oury F.-X. Leenhardt F. Rémésy C. Chanliaud E. Duperrier B. Balfourier F. (2006). Genetic variability and stability of grain magnesium, zinc and iron concentrations in bread wheat. *Eur. J. Agron.* 25 177–185. 10.1016/J.EJA.2006.04.011

[B30] Peleg Z. Cakmak I. Ozturk L. Yazici A. Jun Y. Budak H. (2008). Grain zinc, iron and protein concentrations and zinc-efficiency in wild emmer wheat under contrasting irrigation regimes. *Plant Soil* 306 57–67. 10.1007/s11104-007-9417-z

[B31] Peleg Z. Cakmak I. Ozturk L. Yazici A. Jun Y. Budak H. (2009). Quantitative trait loci conferring grain mineral nutrient concentrations in durum wheat × wild emmer wheat RIL population. *Theor. Appl. Genet.* 119 353–369. 10.1007/s00122-009-1044-z 19407982

[B32] Poland J. A. Brown P. J. Sorrells M. E. Jannink J. L. (2012). Development of high-density genetic maps for barley and wheat using a novel two-enzyme genotyping-by-sequencing approach. *PLoS One* 7:e32253. 10.1371/journal.pone.0032253 22389690 PMC3289635

[B33] Rawat N. Tiwari V. K. Singh N. Randhawa G. S. Singh K. Chhuneja P. (2009). Evaluation and utilization of Aegilops and wild Triticum species for enhancing iron and zinc content in wheat. *Genet. Resour. Crop Evol.* 56 53–64. 10.1007/s10722-008-9344-8

[B34] Roohani N. Hurrell R. Kelishadi R. Schulin R. (2013). Zinc and its importance for human health: an integrative review. *J. Res. Med. Sci.* 18 144–157. 23914218 PMC3724376

[B35] Shi R. Li H. Tong Y. Jing R. Zhang F. Zou C. (2008). Identification of quantitative trait locus of zinc and phosphorus density in wheat (*Triticum aestivum* L.) grain. *Plant Soil* 306 95–104. 10.1007/s11104-007-9483-2

[B36] Singh S. P. Jeet R. Kumar J. Shukla V. Srivastava R. Mantri S. S. (2014). Comparative transcriptional profiling of two wheat genotypes, with contrasting levels of minerals in grains, shows expression differences during grain filling. *PLoS One* 9:111718. 10.1371/journal.pone.0111718 25364903 PMC4218811

[B37] Srinivasa J. Arun B. Mishra V. K. Singh G. P. Velu G. Babu R. (2014). Zinc and iron concentration QTL mapped in a *Triticum spelta* × *T. aestivum* cross. *Theor. Appl. Genet.* 127 1643–1651. 10.1007/s00122-014-2327-6 24865507

[B38] Stevens G. A. Finucane M. M. De-Regil L. M. Paciorek C. J. Flaxman S. R. Branca F. (2013). Global, regional, and national trends in haemoglobin concentration and prevalence of total and severe anaemia in children and pregnant and non-pregnant women for 1995–2011: a systematic analysis of population-representative data. *Lancet Global Health* 1 e16–e25. 10.1016/S2214-109X(13)70001-9 25103581 PMC4547326

[B39] Stoltzfus R. J. Dreyfuss M. L. (1998). *Guidelines for the Use of Iron Supplements to Prevent and Treat Iron Deficiency Anemia.* Washington, DC: ILSI Press.

[B40] Suchowilska E. Wiwart M. Kandler W. Krska R. (2012). A comparison of macro- and microelement concentrations in the whole grain of four Triticum species. *Plant Soil Environ.* 58 141–147. 10.17221/688/2011-PSE

[B41] Tapiero H. Townsend D. Tew K. (2003). Trace elements in human physiology and pathology. copper. *Biomed. Pharmacother.* 57 386–398. 10.1016/S0753-3322(03)00012-X 14652164 PMC6361146

[B42] Tiwari V. K. Rawat N. Chhuneja P. Neelam K. Aggarwal R. Randhawa G. S. (2009). Mapping of quantitative trait loci for grain iron and zinc concentration in diploid a genome wheat. *J. Heredity* 100 771–776. 10.1093/jhered/esp030 19520762

[B43] Uauy C. Distelfeld A. Fahima T. Blechl A. Dubcovsky J. (2006). A NAC gene regulating senescence improves grain protein, zinc, and iron content in wheat. *Science* 314 1298–1301. 17124321 10.1126/science.1133649 PMC4737439

[B44] Wang J. Luo M. C. Chen Z. You F. M. Wei Y. Zheng Y. (2013). *Aegilops tauschii* single nucleotide polymorphisms shed light on the origins of wheat D-genome genetic diversity and pinpoint the geographic origin of hexaploid wheat. *New Phytol.* 198 925–937. 10.1111/nph.12164 23374069

[B45] White P. J. Broadley M. R. (2009). Biofortification of crops with seven mineral elements often lacking in human diets – iron, zinc, copper, calcium, magnesium, selenium and iodine. *New Phytol.* 182 49–84. 10.1111/j.1469-8137.2008.02738.x 19192191

[B46] World Health Organiztion (2006). *Guidelines on Food Fortification With Micronutrients.* Available at: http://apps.who.int/iris/bitstream/handle/10665/43412/9241594012_eng.pdf

[B47] World Health Organiztion (2009). *Mortality and Burden of Disease Attributable to Selected Major Risks.* Available at: http://apps.who.int/iris/bitstream/handle/10665/44203/9789241563871_eng.pdf

[B48] Xu Y. Xu Y. An D. Liu D. Zhang A. Xu H. (2012). Molecular mapping of QTLs for grain zinc, iron and protein concentration of wheat across two environments. *Field Crops Res.* 138 57–62. 10.1016/J.FCR.2012.09.017

[B49] Yu J. Buckler E. S. (2006). Genetic association mapping and genome organization of maize. *Curr. Opin. Biotechnol.* 17 155–160. 10.1016/J.COPBIO.2006.02.003 16504497

[B50] Zhang Z.-L. Ogawa M. Fleet C. M. Zentella R. Hu J. Heo J. O. (2011). Scarecrow-like 3 promotes gibberellin signaling by antagonizing master growth repressor DELLA in Arabidopsis. *Proc. Natl. Acad. Sci. U.S.A.* 108 2160–2165. 10.1073/pnas.1012232108 21245327 PMC3033277

[B51] Zhao F. J. Zhaoa F. J. Sua Y. H. Dunhama S. J. Rakszegib M. Bedob Z. (2009). Variation in mineral micronutrient concentrations in grain of wheat lines of diverse origin. *J. Cereal Sci.* 49 290–295. 10.1016/J.JCS.2008.11.007

[B52] Zhu C. Gore M. Buckler E. S. Yu J. (2008). Status and prospects of association mapping in plants. *Plant Genome J.* 1 5–20. 10.3835/plantgenome2008.02.0089

