# Supplementary figures and images for: Genome-Wide Association Mapping of Grain Micronutrients Concentration in Aegilops tauschii
Source: Front Plant Sci. 2019 Feb 7;10:54. doi: 10.3389/fpls.2019.00054 (PMC6374599; doi:10.3389/fpls.2019.00054)

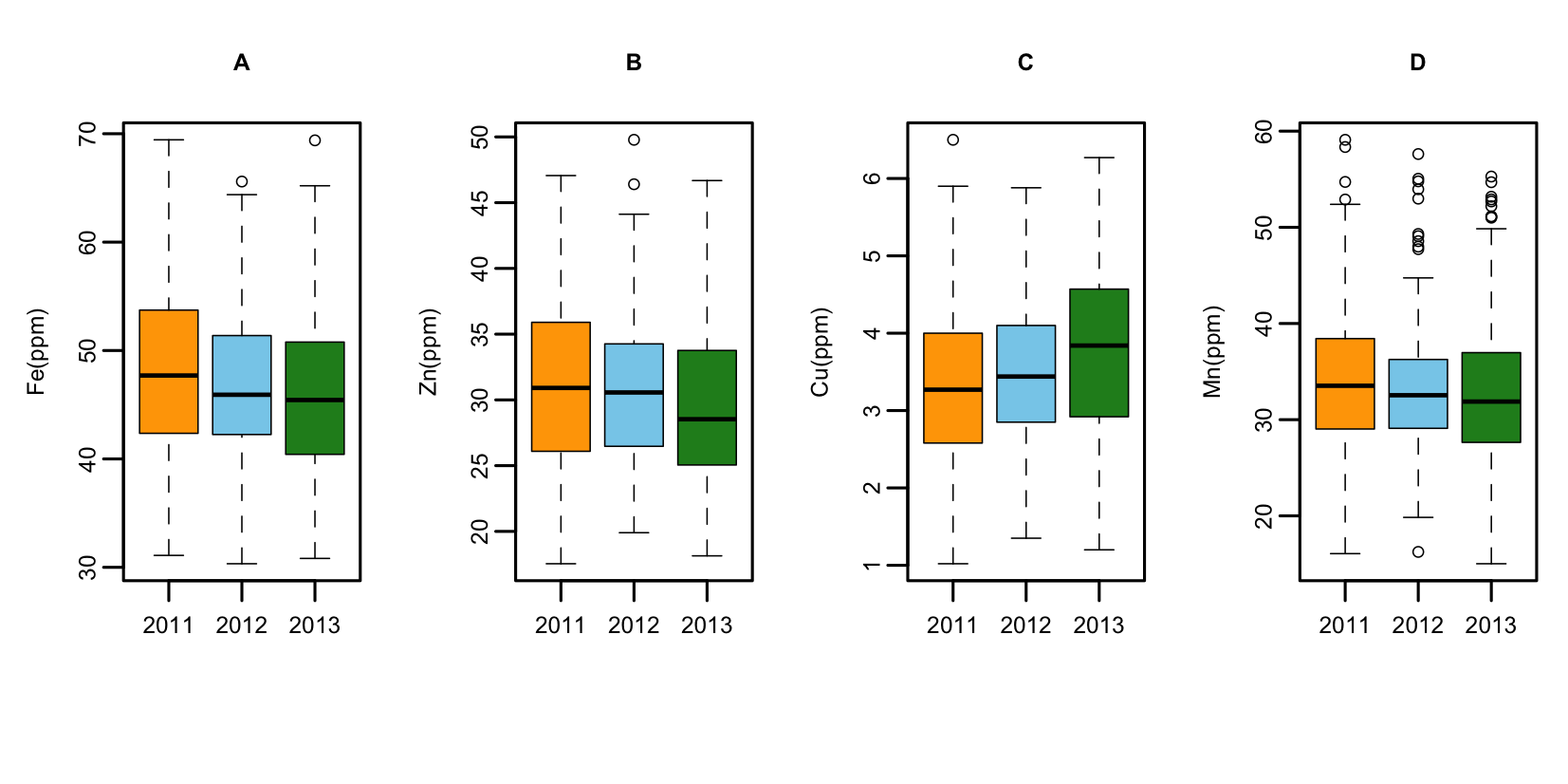

Supplement: Figure S1 — Boxplots representing variation in Ae. tauschii grain micronutrient concentrations for the three crop seasons—2011, 2012, 2013. The variation for grain (A) iron, (B) zinc, (C) copper, and (D) manganese concentrations observed between the years. [file Image_1.TIFF]

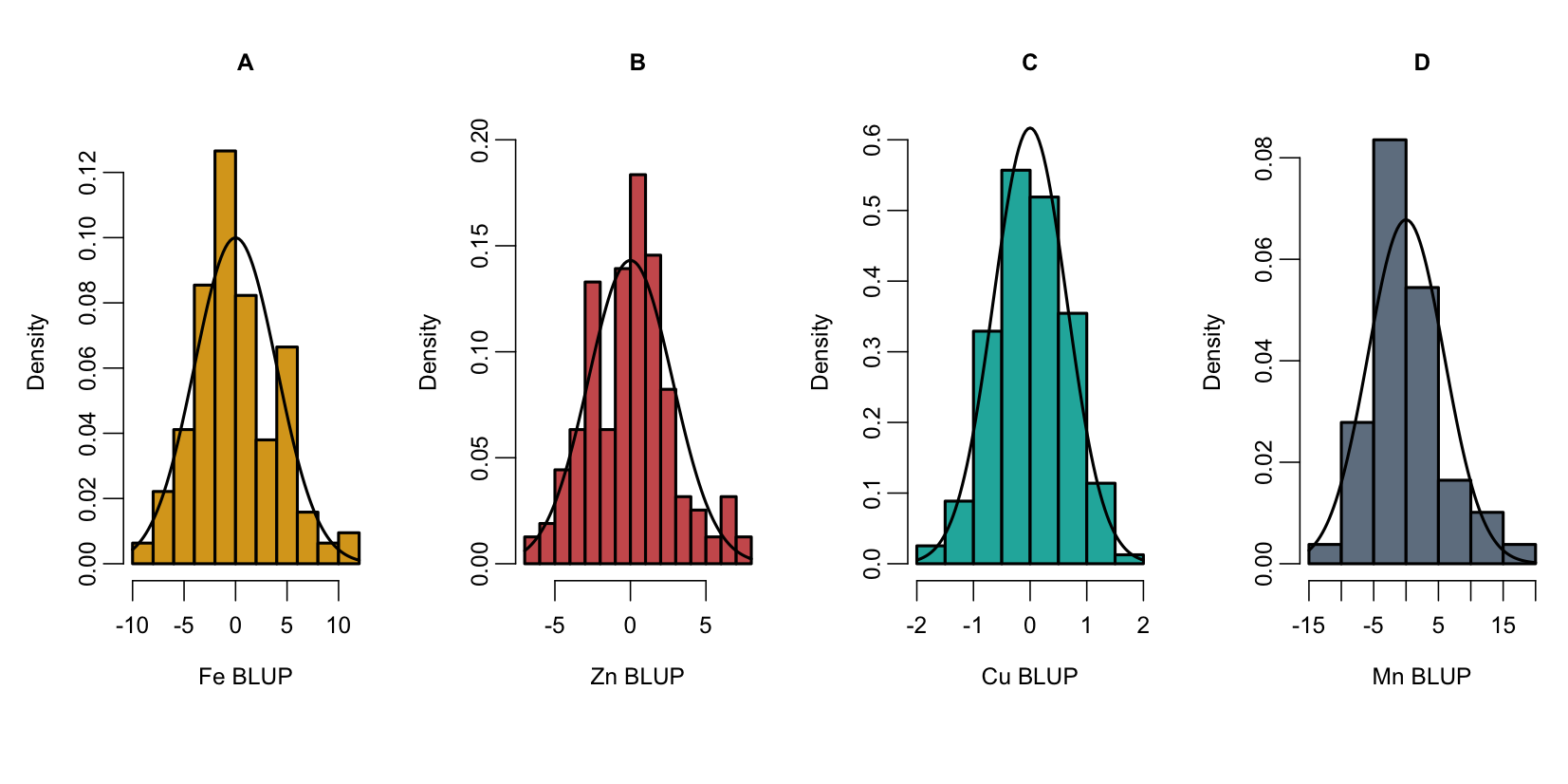

Supplement: Figure S2 — Distribution of best linear unbiased predictions (BLUP) values with normality curve for grain (A) iron, (B) zinc, (C) copper, and (D) manganese. [file Image_2.TIFF]

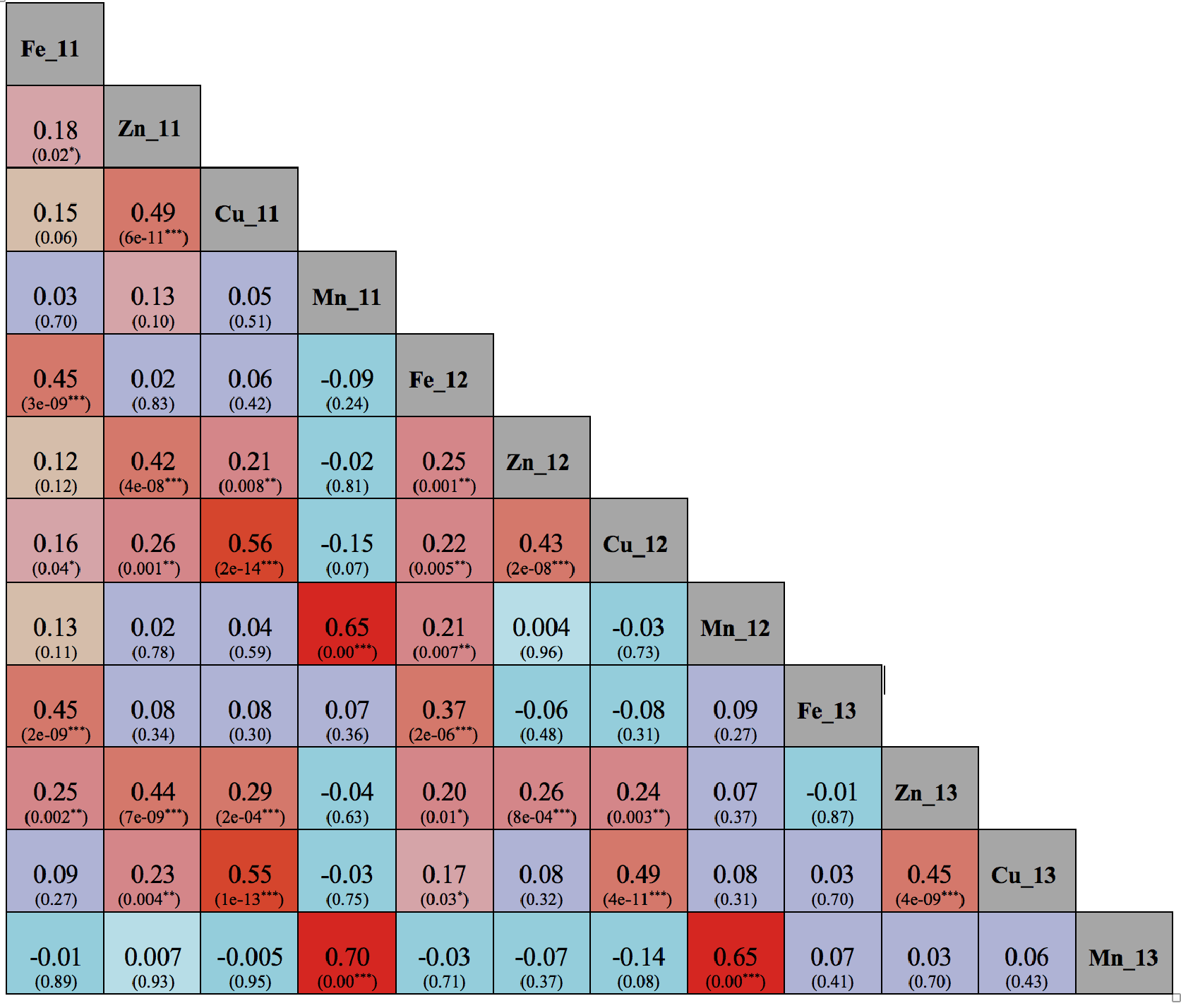

Supplement: Figure S3 — Correlation matrix for grain micronutrient concentrations in Ae. tauschii for year 2011–2013. The values in the column are correlation coefficient at the top and p-value for significant correlations is shown at the bottom. The symbols ∗∗∗, ∗∗, and ∗, indicate significant at p < 0.001, p < 0.01, and p < 0.05, respectively. The colors represent strength of correlation from strongly positive (strong red) to negative (regent blue). [file Image_3.TIF]

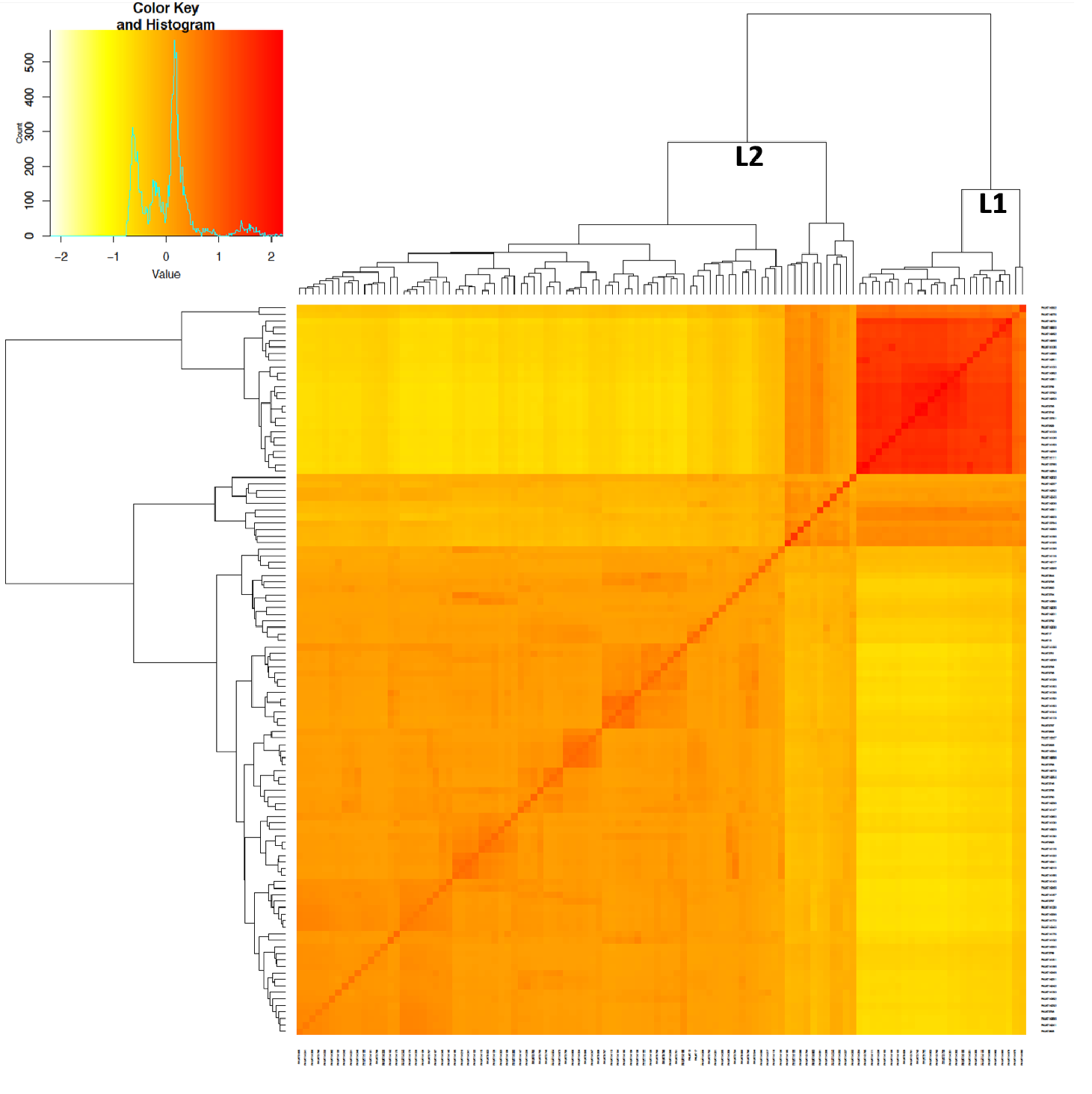

Supplement: Figure S4 — The heat map for non-redundant Ae. tauschii accessions created using GAPIT. The panel is genetically divided into two diverse lineages which are referred to as lineage 1 (L1) and lineage 2 (L1). [file Image_4.TIF]

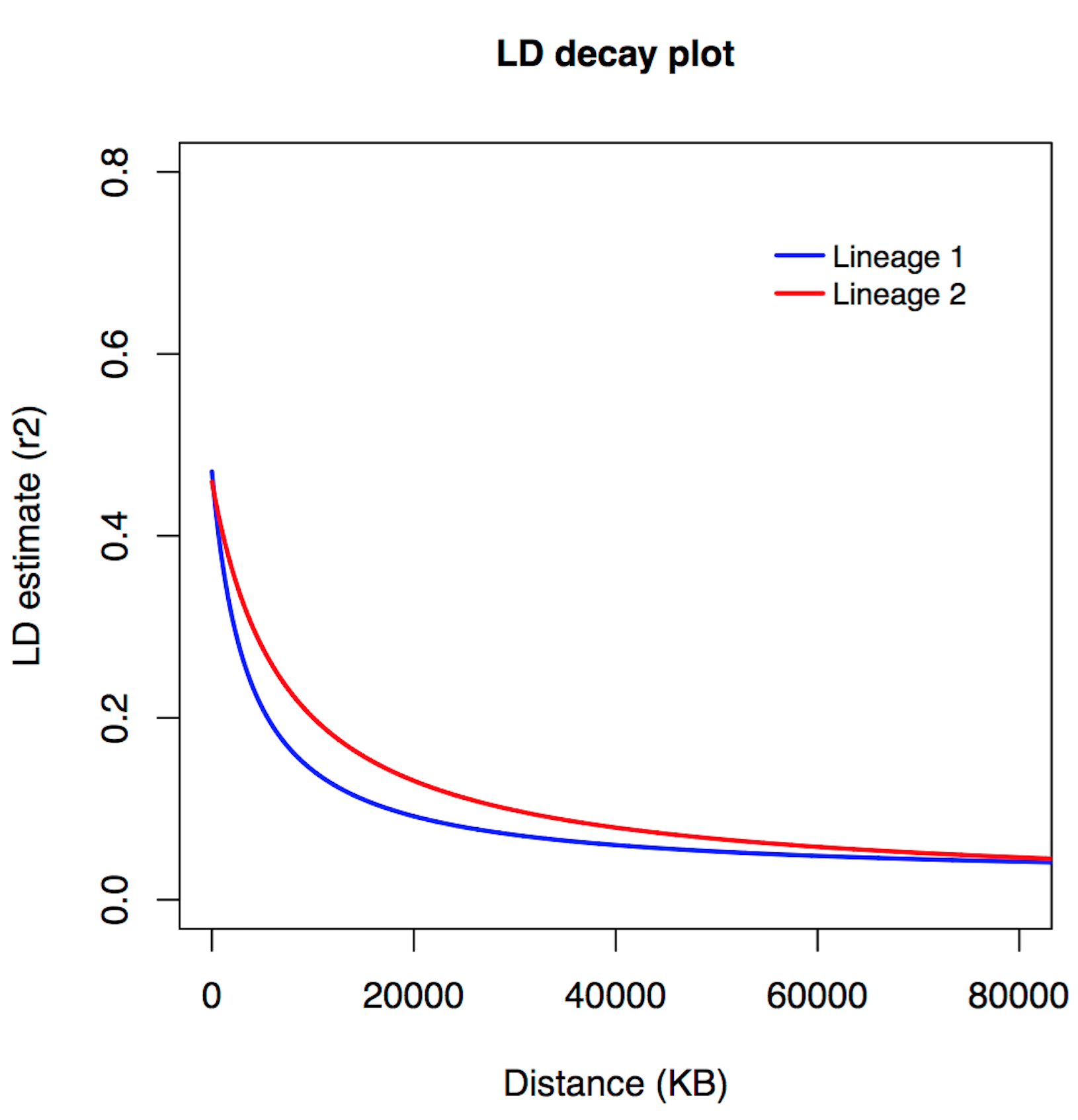

Supplement: Figure S5 — The LD decay plot for lineage 1 and 2 of Ae. tauschii. [file Image_5.TIFF]
