# Supplementary material for: Genome-Wide Association Mapping of Grain Micronutrients Concentration in Aegilops tauschii
Source: Front Plant Sci. 2019 Feb 7;10:54. doi: 10.3389/fpls.2019.00054 (PMC6374599; doi:10.3389/fpls.2019.00054)
Supplement: Table S1 — Comparison of micronutrient concentration between hexaploid wheat cultivars (WL711 and PBW343) and Aegilops tauschii germplasm. [file Table_1.pdf]

Supplementary Table S1 Comparison of micronutrient concentration between hexaploid wheat cultivars (WL711 and PBW343) and *Aegilops tauschii* germplasm

**Micronutrient**	**Concentration (ppm)**		
	PBW343 (Mean)	WL711 (Mean)	Range in *Ae. tauschii* germplasm
Iron	38.95	35.05	30.33 – 69.44
Zinc	24.60	23.80	17.54 – 49.78
Copper	2.35	1.18	1.02 – 6.50
Manganese	33.60	21.69	15.02 – 59.10
