# Supplementary material for: Genome-Wide Association Mapping of Grain Micronutrients Concentration in Aegilops tauschii
Source: Front Plant Sci. 2019 Feb 7;10:54. doi: 10.3389/fpls.2019.00054 (PMC6374599; doi:10.3389/fpls.2019.00054)
Supplement: Table S2 — Descriptive statistics of Ae. tauschii accessions for micronutrients concentration based on two lineages (L1, L2). [file Table_2.pdf]

Supplementary Table S2 Descriptive statistics of *Ae. tauschii* accessions for micronutrients concentration based on two lineages (L1, L2)

**Traits**	**Lineage 1 (L1)**			**Lineage 2 (L2)**		
	**Mean** (ppm)	**S.D.**	**C.V%**	**Mean** (ppm)	**S.D.**	**C.V%**
Iron	44.81	6.50	15.00	47.72	7.26	16.00
Zinc	29.84	6.02	22.00	30.66	5.45	18.00
Copper	3.26	0.90	27.00	3.71	1.06	30.00
Manganese	37.23	9.95	26.00	31.65	5.85	17.00
